# Association of c-Raf expression with survival and its targeting with antisense oligonucleotides in ovarian cancer

**DOI:** 10.1054/bjoc.2001.2139

**Published:** 2001-11

**Authors:** F McPhillips, P Mullen, B P Monia, A A Ritchie, F A Dorr, J F Smyth, S P Langdon

**Affiliations:** ICRF Medical Oncology Unit, Western General Hospital, Edinburgh, EH4 2XU, UK; ISIS Pharmaceuticals Inc, Carlsbad, CA, USA

**Keywords:** Raf, antisense, ovarian, cancer, oligodeoxynucleotide

## Abstract

c-Raf is an essential component of the extracellular related kinase (ERK) signal transduction pathway. Immunohistochemical staining indicated that c-Raf was present in 49/53 ovarian adenocarcinomas investigated and high c-Raf expression correlated significantly with poor survival (*P* = 0.002). c-Raf protein was detected in 15 ovarian cancer cell lines. Antisense oligodeoxynucleotides (ODNs) (ISIS 5132 and ISIS 13650) reduced c-Raf protein levels and inhibited cell proliferation in vitro. Selectivity was demonstrated by the lack of effect of ISIS 5132 on A-Raf or ERK, while a random ODN produced only minor effects on growth and did not influence c-Raf expression. ISIS 5132 produced enhanced apoptosis and cells accumulated in S and G _2_/M phases of the cell cycle. In vivo, ISIS 5132 inhibited growth of the s.c. SKOV-3 xenograft while a mismatch ODN had no effect. These data indicate that high levels of c-Raf expression may be important in ovarian cancer and use of antisense ODNs targeted to c-Raf could provide a strategy for the treatment of this disease. © 2001 Cancer Research Campaign http://www.bjcancer.com


*British Journal of Cancer* (2001) **85**(11), 1753–1758
© 2001 Cancer Research Campaign
doi: 10.1054/ bjoc.2001.2139, available online at http://www.idealibrary.com on IDEAL

http://www.bjcancer.com

### Association of c-Raf expression with survival and its targeting with antisense oligonucleotides in ovarian cancer

**F McPhillips[1], P Mullen[1], BP Monia[2], AA Ritchie[1], FA Dorr[2], JF Smyth[1] and SP Langdon[1]**

[1]ICRF Medical Oncology Unit, Western General Hospital, Edinburgh EH4 2XU, UK; [2]ISIS Pharmaceuticals Inc, Carlsbad, CA, USA

**Summary** c-Raf is an essential component of the extracellular related kinase (ERK) signal transduction pathway. Immunohistochemical staining indicated that c-Raf was present in 49/53 ovarian adenocarcinomas investigated and high c-Raf expression correlated significantly with poor survival ($P = 0.002$). c-Raf protein was detected in 15 ovarian cancer cell lines. Antisense oligodeoxynucleotides (ODNs) (ISIS 5132 and ISIS 13650) reduced c-Raf protein levels and inhibited cell proliferation in vitro. Selectivity was demonstrated by the lack of effect of ISIS 5132 on A-Raf or ERK, while a random ODN produced only minor effects on growth and did not influence c-Raf expression. ISIS 5132 produced enhanced apoptosis and cells accumulated in S and $G_2$/M phases of the cell cycle. In vivo, ISIS 5132 inhibited growth of the s.c. SKOV-3 xenograft while a mismatch ODN had no effect. These data indicate that high levels of c-Raf expression may be important in ovarian cancer and use of antisense ODNs targeted to c-Raf could provide a strategy for the treatment of this disease. © 2001 Cancer Research Campaign http://www.bjcancer.com

**Keywords**: Raf; antisense; ovarian; cancer; oligodeoxynucleotide c-Raf is a ubiquitously expressed 74 kDa serine/threonine protein kinase which is central to the ERK pathway (Daum et al, 1994) and has long been implicated in oncogenesis (Rapp et al, 1988; Storm et al, 1990). Despite this, little information on c-Raf protein expression in ovarian cancer is available. c-Raf is activated by phosphorylation after translocation to the plasma membrane by a mechanism that involves Ras (Avruch et al, 1994). Mutations in *ras* genes have been frequently detected in ovarian tumours and Ras protein is downstream of receptors such as the EGF/erbB receptor tyrosine kinases (Rozakis-Adcock et al, 1993; Teneriello et al, 1993). Compared to normal ovary and benign ovarian tumours, malignant ovarian tumours have increased expression of EGF receptor (erbB-1) (Stewart et al, 1992) and erbB2 (Meden and Kuhn, 1997). The erbB receptors and Ras are currently being investigated as potential targets for growth inhibitors in cancer (Cowsert, 1997; Fan and Mendelsohn, 1998; Witters et al, 1999). Targeting growth inhibitors to c-Raf in cancer cells would eliminate c-Raf-mediated cell proliferation signals from these upstream effectors and prevent cell proliferation arising from over-expression of c-Raf.

Targeting genes using antisense technology provides a highly selective, sequence-specific mechanism for inhibiting the expression of a chosen gene product. A number of antisense oligodeoxynucleotides (ODNs) are currently undergoing clinical trials for the treatment of cancer (Cho-Chung, 1999; Nemunaitis et al, 1999; Yuen et al, 1999). ISIS 5132, a 20-mer phosphorothioate antisense ODN (first generation compound) that targets the 3′ untranslated region of c-Raf mRNA, inhibits the expression of c-Raf mRNA and protein in lung, colon, cervical and prostate cancer cells (Monia et al, 1996a; Lau et al, 1998). Second generation antisense ODNs containing 2′-methoxyethyl sugar modifications, such as ISIS 13650, have also been developed and these may be more potent inhibitors of mRNA expression (Monia, 1997).

To investigate the role and function of c-Raf in ovarian cancer, we have measured c-Raf expression in primary tumours and ovarian cancer cell lines and studied relationships between protein expression and histology, grade of differentiation, tumour stage and patient survival time. To explore the therapeutic potential of first- and second-generation c-Raf antisense ODNs in ovarian cancer, the extent of growth inhibition following antisense ODN treatment was investigated in ovarian cancer cell lines and a xenograft model. Our findings demonstrate that high levels of c-Raf expression are important in ovarian cancer and use of antisense ODNs targeted to c-Raf may provide a novel strategy for the treatment of this disease.

### MATERIALS AND METHODS

### Tumour samples

Fresh primary ovarian tumour tissue was obtained from 53 previously untreated patients with epithelial ovarian cancer at initial debulking surgery, transferred to liquid nitrogen, then formalin-fixed and embedded in paraffin. Tumour histology was assessed on paraffin embedded sections and classified according to WHO criteria (details in Table 1).

### Cell lines

PEO1, PEO1$^{CDDP}$, PEO4, PEO6, PEO14 and PEO16 were established as described previously (Langdon et al, 1988); SKOV-3 and CaOV3 cells were obtained from the American Type Culture

*Received 6 August 2001*
*Accepted 31 August 2001*

*Correspondence to:* SP Langdon

Collection (Manassas, VA, USA); OVCAR3, OVCAR4 and OVCAR5 were obtained from Dr TC Hamilton (Fox Chase Institute, Philadelphia, PA, USA); 41M, 59M, OAW42 and A2780 cells were obtained from the European Tissue Collection (Porton Down). All cell lines were routinely grown as monolayer cultures in RPMI 1640 (Life Technologies Inc, Gaithersburg, MD) supplemented with 10% heat-inactivated fetal calf serum and 100 iu $ml^{-1}$ penicillin/streptomycin. Cells were maintained at 37˚C in a 5% $CO_2$ humidified incubator.

### Immunohistochemistry

Sections (3 μm) were deparaffinised and rehydrated. Endogenous peroxidase activity was blocked by incubating sections in 3% $H_2O_2$ for 30 min immersing in citric acid buffer (0.005M, pH 6.0) and microwaving for $3 \times 5$ min. Slides were washed in 0.05M Tris/HCI buffer (pH 7.6) then incubated in 20% fetal calf serum in the above Tris buffer for 10 min. Anti-c-Raf antibody (R19120, Transduction Laboratories) was used at 1:10–1:20 dilution in 20% fetal calf serum and sections were incubated for 1.5–2 h. A streptavidin–biotin multilink method (StrAviGen Multilink kit; Biogenex, San Ramon, CA) was used to detect reactivity. Sections were stained with a secondary multilink antibody at a 1:20 dilution for 30 min, then incubated with a horseradish-peroxidase-labelled streptavidin complex at a 1:20 dilution for 30 min.

Diaminobenzidine tetrachloride was used as chromagen and applied for 5 min. Sections were lightly counterstained in haematoxylin, dehydrated and mounted. Negative controls for each tumour section were included by replacing the primary antibody with Tris buffer. Immunoreactive scores between 0 and 12 were generated for each sample and represent the product of intensity (0 = negative, 1 = weak, 2 = moderate, 3 = strong) and percentage positive cell staining (0 = 0%, 1 = 1–25%, 2 = 26–50%, 3 = 51–75%, 4 = 76–100%).

### Statistics

Relationships between variables were analysed using the Fisher's exact test, the student *t*-test and the Mann–Whitney test where appropriate. Differences in survival were determined using the Kaplan–Meier method and groups were compared using the log-rank test and $\chi^2$ test.

### Western blotting

Cells were grown to 70% confluence, washed twice with PBS, and lysed in ice cold hypotonic lysis buffer (50 mM Tris-HCI (pH 7.5), 5 mM EGTA (pH 8.5), 150 mM NaCl, 1% Triton X-100, 2 mM sodium orthovanadate, 50 mM sodium fluoride, 1 mM phenylmethanesulfonylfluoride, 10 μg $ml^{-1}$ leupeptin, 10 μg $ml^{-1}$ aprotinin and 10 mM sodium molybdate). Lysates were centrifuged for 6 min at 13 000 rpm in a microfuge. Tumour samples (100 mg) were finely chopped, then homogenized on ice in a Silverson homogenizer in 1.8 ml lysis buffer (excluding Triton X-100). Samples were incubated on ice after the addition of 1% Triton X-100, then centrifuged 14 000 rpm for 30 min. Protein concentrations of supernatants were determined using the Bio-rad Protein Assay Kit (Bio-rad, Richmond, CA). Cell lysates (30 μg) or tumour lysates (50 μg) were resolved on 10% or 12% SDS-PAGE then transferred electrophoretically overnight onto Immobilon-P membranes (Millipore, Bedford, MA). After transfer, membranes were blocked with 1% blocking agent in TBS (20 mM Tris-HCl, 137 mM NaCl, pH 7.5) before probing with the appropriate primary antibody, anti-c-Raf (R19120, Transduction Laboratories, Lexington, KY), anti-A-Raf (R14320, Transduction Laboratories), anti-ERK (E16220, Transduction Laboratories) overnight at 4˚C. Immunoreactive bands were detected using enhanced chemiluminescent reagents (1520709, Boehringer Mannheim) and Hyperfilm ECL (Amersham, Buckinghamshire, UK).

### Antisense ODNs

Antisense ODNs targeted to the 3′ untranslated region of c-Raf mRNA (sequence: TCCCGCCTGTGACATGCATT) were supplied by ISIS Pharmaceuticals (Carlsbad, CA). Two forms of the antisense ODNs were used: a first generation compound (ISIS 5132) which has a phosphorothioate backbone and a second generation compound (ISIS 13650) which also has a phosphorothioate backbone and the addition of 2′ methoxyethyl groups on the sugar moiety. 2 control ODNs were available: a second generation random ODN (ISIS 16971; sequence, TCACATTGGCGCTTAGCCGT) and a first generation mismatch ODN (ISIS 10353 sequence, TCCCGCGCACTTGATGCATT).

### Growth and protein inhibition experiments

For growth inhibition experiments, log phase cells were trypsinized and seeded into 24-well tissue culture plates ($1 \times 10^4$ in 1 ml) and incubated to reach 40–60% confluence. Cells were then washed with PBS before adding 250 μl of Optimem (Gibco-BRL) containing 'Lipofectin' (Gibco-BRL) (6 μl $ml^{-1}$). Antisense and random ODNs were added (50 nM–200 nM) from 50 μM stock solutions. Cells were incubated at 37˚C for 3 h, washed with PBS, replenished with RPMI (plus 10% fetal calf serum and 100 iu $ml^{-1}$ penicillin/streptomycin) and replaced in the incubator for the remainder of the time course. Cells were trypsinized and counted at the appropriate time point using a 'ZM' Coulter Counter.

c-Raf protein inhibition experiments were carried out as above except that cells ($2.5 \times 10^5$ in 4 ml) were plated into 60 mm diameter petri dishes and washed with PBS (2 ml) prior to addition of Optimem/Lipofectin/ODN (1 ml). Cells were lysed and analysed by Western blotting as previously described.

### Cell cycle analysis

DNA analysis of treated cells was carried out on a Becton Dickinson 'FACSCalibur' flow cytometer using methodology described by Levack et al (1987).

### Apoptosis assay

SKOV-3 cells were treated with ODNs as described above and apoptosis was measured using the TACS Annexin V-FITC kit (R& D Systems) following the prescribed protocol.

### Xenograft experiments

Female adult nude (*nu/nu*) mice were obtained from ICRF (Clare Hall, South Mimms, UK) and maintained in negative pressure isolators. SKOV-3 cells ($5 \times 10^6$ cells/injection) were injected into both flanks of 2 groups of mice. The control group consisted of 10 mice (2 tumours/mouse) and the treatment groups

 © 2001 Cancer Research Campaign consisted of 5 mice. ODNs were administered i.p. daily in PBS on the days indicated. Tumour volumes, assessed twice weekly by caliper measurements of the tumour in 2 dimensions, were calculated by the formula $\pi/6 \times \text{length} \times \text{width}^2$. Treatment was initiated either 24 h after cell implantation or when tumours had reached a median diameter of 4 mm. Relative tumour volume was evaluated by dividing the volume at day × (the number of days after the start of treatment) by the volume at day 0 (the day treatment started). UKCCCR Guidelines (1998) were followed throughout.

### RESULTS

### c-Raf expression in primary ovarian cancer c-Raf expression was identified in 49 of 53 ovarian cancer sections and varied from weak to intense staining (examples are illustrated in Figure 1A). Immunoreactivity was found almost exclusively in the epithelial cells with only minor staining in the stroma. The relationships between immunoreactive scores and clinical and pathological parameters are represented in Table 1. Serous adenocarcinomas expressed higher levels of c-Raf than all other subtypes combined ($P$ = 0.005, Fisher's exact test). No significant associations between c-Raf expression level and either stage (I / II vs III / IV, $P$ = 0.18 Fisher's exact test) or grade of differentiation (poor vs moderate/well, $P$ = 0.17, Fisher's exact test) were observed.

Survival data were available for 52/53 patients. High c-Raf expression was linked with poor survival in this group (Figure 1B). The survival of patients whose tumours had an immunoreactive score ≥ 7 ($n$ = 14) was significantly poorer than that of patients with scores ≤ 6 ($n$ = 38) ($P$ = 0.002, log-rank test). Since histology and stage are major prognostic variables, we also analysed survival in patients having stage III serous adenocarcinomas which represented a majority sub-group ($n$ = 28). Again patients whose tumours had a higher level of c-Raf expression (immunoreactive score of ≥ 7) ($n$ = 12) had significantly poorer survival ($P$ = 0.035, log-rank test) than patients with tumours expressing lower levels of c-Raf ($n$ = 16).

**Table 1** c-Raf expression in primary ovarian cancer

| Immunoscore | Number of patients with immunoscore[a] 0–6 | 7–12 | $P$ value |
|---|---|---|---|
| All tumours | 39 | 14 | |
| Stage | | | |
| I / II | 13 | 2 | 0.18[b] |
| III / IV | 23 | 12 | |
| Differentiation | | | |
| Well / moderate | 14 | 2 | 0.17[b] |
| Poor | 21 | 11 | |
| Histology | | | |
| Serous | 16 | 12 | |
| Endometrioid | 17 | 1 | 0.005[c] |
| Clear cell | 5 | 1 | |
| Mucinous | 1 | 0 | |

The expression of c-Raf was determined by immunohistochemical staining as described in Materials and Methods and scores between 0 and 12 were generated for each sample. [a]53 patient samples were analysed for expression but information on stage and grade were available for 50 and 48 patients respectively. [b]Fisher's exact test. [c]Fisher's exact test (serous vs rest).

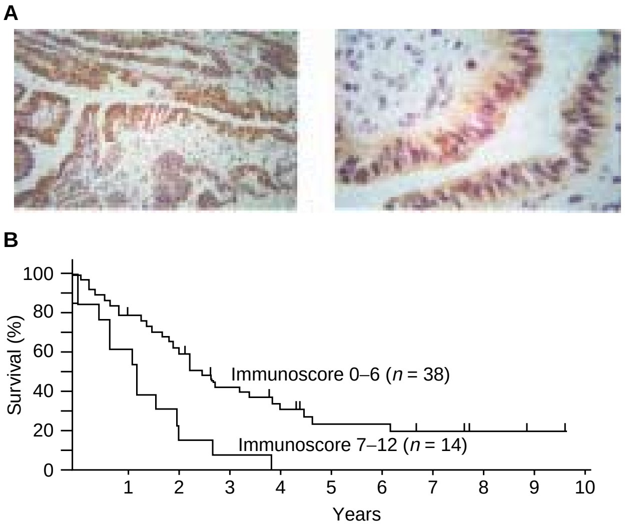

**Figure 1** High c-Raf expression is associated with poor survival in ovarian cancer. (**A**) Examples of c-Raf immunoreactivity, detected as described in 'Materials and Methods'. Epithelial cells are diaminobenzidine positive (brown) and counterstain is hematoxylin (blue). Low and high power magnifications. (**B**) Kaplan–Meier survival curves with log-rank analysis; high expressers vs low expressers ($P$ = 0.002)

### c-Raf expression in ovarian cancer cell lines c-Raf protein, detected by Western blotting, was evident in all 15 cell lines analysed. Levels of c-Raf varied 24-fold between cell lines, compared to only a 2.5-fold and 11-fold variation in ERK and A-Raf respectively (Figure 2A and 2B).

### Antisense oligonucleotides reduce c-Raf protein levels in SKOV-3 cells

The ability of the antisense ODNs ISIS 13650 and ISIS 5132 to selectively reduce the amount of c-Raf protein was investigated in

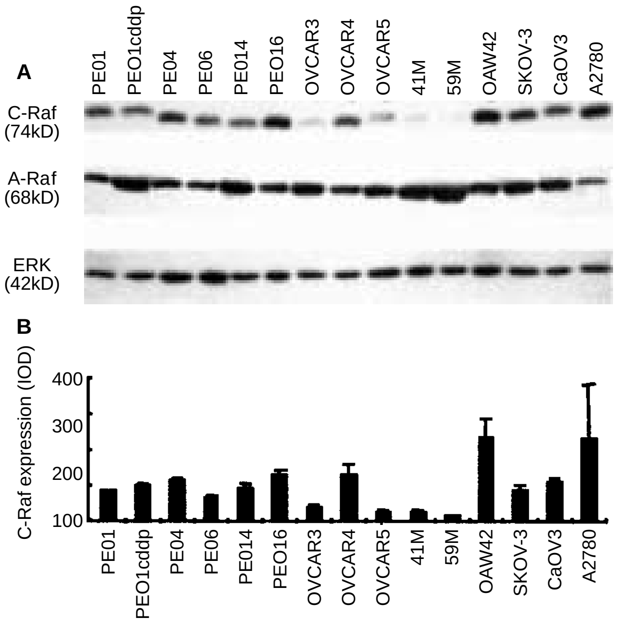

**Figure 2** (**A**) Expression of c-Raf, A-Raf and ERK2 in 15 ovarian cancer cell lines. Cells were lysed and samples electrophoresed as described in 'Materials and Methods'. Blots were probed with anti-c-Raf, anti-A-Raf or anti-ERK2. Data shown is a typical result of $n$ = 3. (**B**) Densitometric analysis (Integrated Optical Density units) of c-Raf expression, $n$ = 3

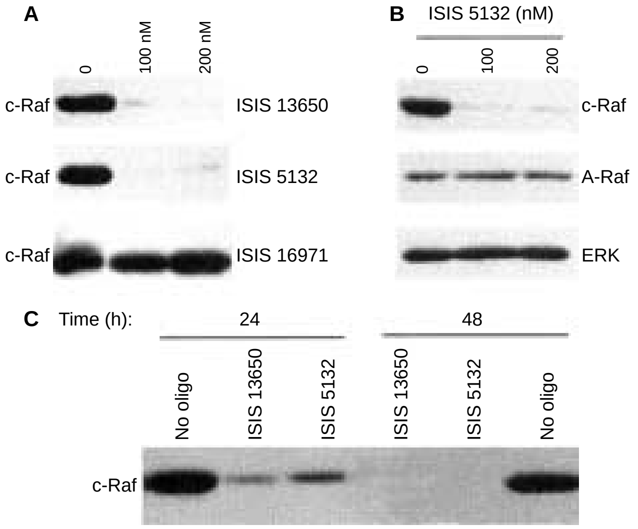

**Figure 3** Effect of c-Raf antisense ODNs on c-Raf protein level in SKOV-3 cells. After treatment with ISIS 5132, ISIS 13650 or ISIS 16971, cells were lysed and samples electrophoresed and probed with anti-c-Raf, anti-A-Raf or anti-ERK antibodies as described in 'Materials and Methods'. (**A**) Suppression of c-Raf protein by c-Raf antisense oligonucleotides but not a random ODN. (**B**) Suppression of c-Raf but not A-Raf and ERK by ISIS 5132 at 48 h after treatment. (**C**) Effect of time of c-Raf protein reduction by ISIS 5132 and ISIS 13650

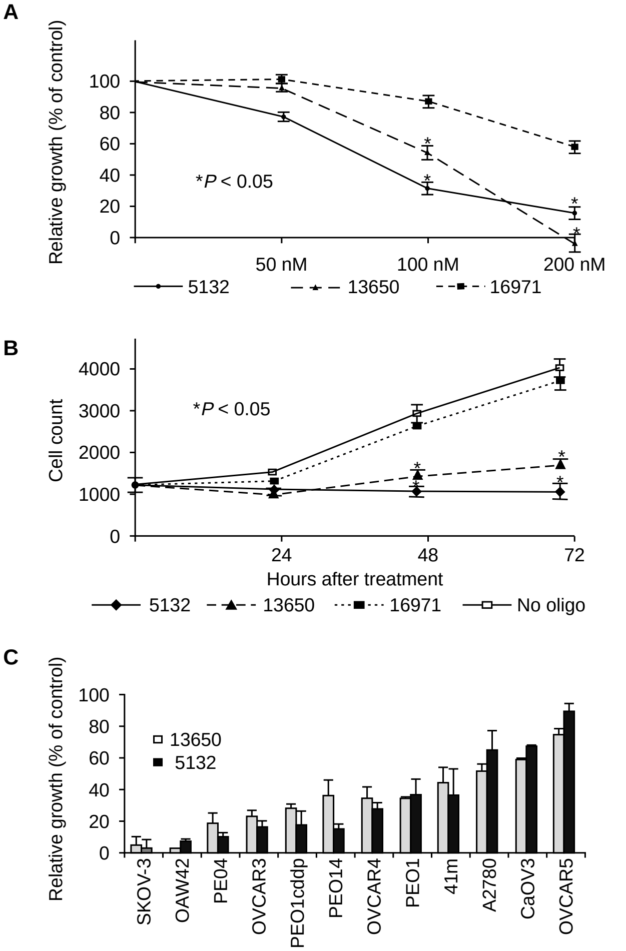

**Figure 4** C-Raf antisense ODNs inhibit growth in ovarian cancer cell lines. (**A**) 72 h after treatment with ISIS 5132, ISIS 13650 or ISIS 16971 SKOV-3 cells were trypsinized and counted on a 'ZM' Coulter Counter. Cell counts taken at day 0 (the day treatment began) are deducted from the 72 h cell count. The value at each concentration is expressed as the percentage growth compared to 100%, the value obtained from untreated cells grown for 72 h. (**B**) SKOV-3 cells were counted at 24, 48 and 72 h after treatment with ISIS 5132, ISIS 13650 or ISIS 16971. (**C**) 72 h after treatment with ISIS 5132 or ISIS 13650 cells were trypsinized and counted. In all cell lines except OVCAR5 growth rates of treated cells are significantly different from untreated cells ($P < 0.05$; student *t*-test)

SKOV-3 cells. After 48 h, both ODNs reduced c-Raf levels at 100 nM and 200 nM (Figure 3A). In comparison, similar concentrations of the random control ODN ISIS 16971 had no effect on c-Raf levels. The specificity of ISIS 5132 for c-Raf was examined by measuring its effects on A-Raf and ERK and after 48 h, ISIS 5132 (200 nM) had no effect on the expression of either of these proteins (Figure 3B). To determine the time required for c-Raf antisense ODNs to exert their effects on c-Raf protein, the level of c-Raf present in SKOV-3 cells was assayed at various intervals following antisense treatment. Both ISIS 13650 (200 nM) and ISIS 5132 (200 nM) reduced c-Raf levels by > 50% at 24 h and almost complete removal of c-Raf protein was observed at 48 h (Figure 3C).

### c-Raf antisense ODNs inhibit cellular proliferation in SKOV-3 cells

To evaluate the effectiveness of c-Raf antisense ODNs as anti-proliferative agents, SKOV-3 cells were treated with ISIS 13650, ISIS 5132 or random ODN and the cell number determined 72 h after treatment. At 200 nM both ISIS 13650 and ISIS 5132 demonstrated > 80% inhibition of cell growth, compared to <30% inhibition shown by the random ODN (Figure 4A). The time required for c-Raf antisense ODNs to exert their effects on cell proliferation was investigated in SKOV-3 cells. Cells not exposed to ODN (control cells) and cells treated with mismatch ODN (200 nM) grew steadily over the time course demonstrating a 3-fold increase in cell number over the 72 h period (Figure 4B). In contrast, the addition of 200 nM ISIS 5132 or ISIS 13650 resulted in marked growth inhibition at all time points investigated, with both antisense ODNs preventing cell growth in the first 24 h after treatment (Figure 4B).

### Effect of c-Raf antisense ODNs in a panel of ovarian cancer cell lines

The ability of c-Raf antisense ODNs to inhibit cell growth was further investigated in a panel of 12 ovarian cancer cell lines 48 h after treatment. Both ISIS 13650 and ISIS 5132 inhibited cell proliferation by ≥ 60% in 9/12 cell lines examined (Figure 4C) compared to < 30% inhibition shown by the random ODN in selected cell lines.

### ISIS 5132 causes cell cycle arrest and enhanced apoptosis in SKOV-3 cells

DNA analysis showed an accumulation of cells arrested in the S and $G_2$/M phases of the cell cycle with a concomitant reduction in the $G_0$/$G_1$ phase. These specific effects were dose-dependent and not seen in either untreated cells or cells treated with ISIS 16971 (Figure 5A). A dose-dependent enhancement of apoptosis was

*© 2001 Cancer Research Campaign*

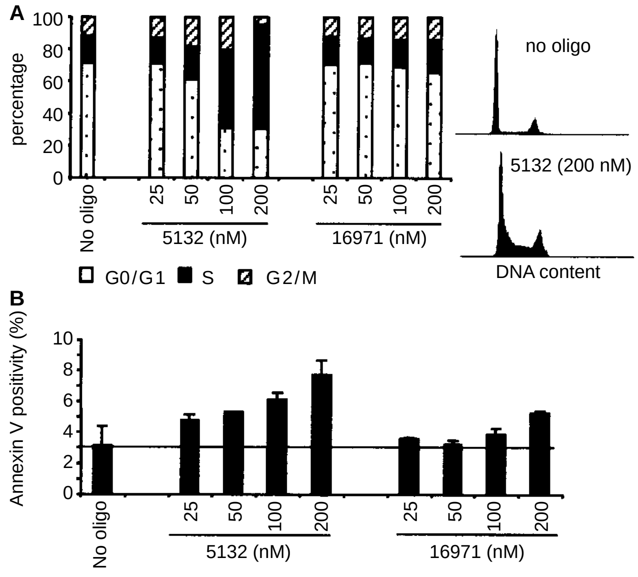

**Figure 5** Effect of c-Raf antisense ODNs on cell cycle distribution and apoptosis in SKOV-3 cells. (**A**) 48 h after treatment with ISIS 5132 or ISIS 16971 cells were trypsinized and stained for total DNA content using propidium iodide. Cell cycle distribution was carried out using a 'FACSCalibur' flow cytometer and results analysed using 'Modfit' software. The relative proportion of cells in the G0/G1, S and G2/M phase of the cycle is shown along with representative DNA histograms. (**B**) Apoptosis levels were also assayed after 48 h in similarly treated SKOV-3 cells that had been exposed to FITC-labelled annexin V and counterstained with propidium iodide similarly seen in cells treated with ISIS 5132 compared with untreated cells or cells exposed to ISIS 16971 (Figure 5B).

### ISIS 5132 inhibits growth of SKOV-3 ovarian cancer xenografts

ISIS 5132 inhibited the growth of SKOV-3 cells implanted as a subcutaneous xenograft in the flanks of nude mice. Treatment initiated 24 h after implantation of cells reduced the growth rate of these tumours at both 10 and 25 mg $kg^{-1}$ $day^{-1}$ compared to a vehicle control (Figure 6A). A mismatch control (ISIS 10353) was available for these studies and had no effect on growth. When treatment was delayed until tumours had reached a median size of 4 mm, ISIS 5132 (25 mg $kg^{-1}$ $day^{-1}$) again produced a significant effect on growth (Figure 6B).

### DISCUSSION

In the present study, which is the first to look at the prognostic significance of c-Raf expression in ovarian cancer, we show that high c-Raf expression is a negative prognostic factor. c-Raf was present in 92% of the ovarian adenocarcinomas investigated and a high level of c-Raf expression correlated significantly with both poor survival and serous histology. Within the serous subset, there was again a significant correlation between expression and survival, indicating that high c-Raf expression is associated with poor survival irrespective of other parameters such as histology and stage. The epithelial tumour cells were highly positive for c-Raf staining compared to stromal cells, which showed little c-Raf expression.

The c-Raf signalling pathway plays an important role in the growth regulation of some ovarian cancers. ERK activation has

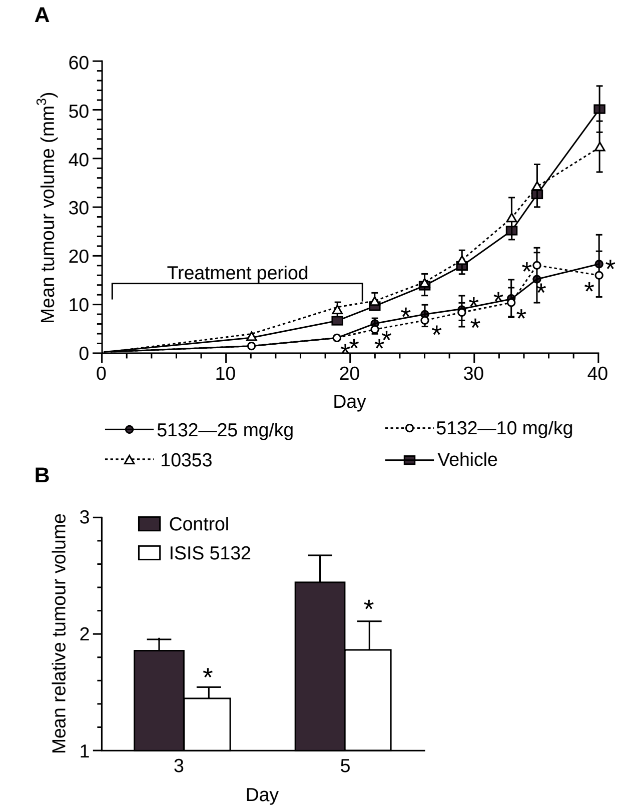

**Figure 6** Effect of ISIS 5132 on in vivo growth of SKOV-3 cells. (**A**) Treatment initiated 24 h after implantation of $5 \times 10^6$ cells s.c. ISIS 5132 was administered i.p. at 25 or 10 mg $kg^{-1}$ $day^{-1}$ for 21 days and compared with a mismatch control (ISIS 10353–25 mg $kg^{-1}$ $day^{-1}$) or vehicle only. (**B**) ISIS 5132 treatment of advanced SKOV-3 tumours. Tumours were allowed to grow for 35 days before treatment (mean diameter = 4 mm). Relative tumour volumes after 3 and 5 days treatment are shown. Mean ± standard errors of at least 10 tumours indicated. *significantly different from vehicle, $P < 0.05$ (*t*-test)

previously been identified in ovarian cancer cell lines and attributed to an increase in MEK and c-Raf activity (Hoshino et al, 1999). In addition, ERK activation has been shown to correlate linearly with increasing concentrations of MEK (Zheng and Guan, 1993). These data in combination with our finding that c-Raf is highly expressed in most of the ovarian cancer cell lines suggest that an increase in c-Raf level may contribute to increased signalling through the ERK pathway. Further analysis of the ERK signalling pathway in ovarian cancer cell lines is essential to elucidate the role of c-Raf in this disease, and will form the basis of future studies.

The use of antisense ODNs has allowed us to establish that c-Raf plays an important role in the proliferation of ovarian cancer cell lines, supporting conclusions of other studies that c-Raf is a critical mediator of oncogenic transformation (Storm et al, 1990; Daum et al, 1994). We have shown that both ISIS 5132 and ISIS 13650 reduce c-Raf protein in SKOV-3 ovarian cancer cells, in line with observations in other disease types (Monia et al, 1996a; Monia, 1997; Lau et al, 1998). Evidence to support specificity was provided by the observations that while c-Raf was reduced, related signalling molecules were unaffected and a random ODN (ISIS 16971) had no effect on c-Raf protein expression.

Investigation of the effects of antisense ODNs in a panel of ovarian cancer cell lines has allowed an insight into the efficiency of these antisense compounds in a range of cell types with variable amounts of c-Raf protein. Despite a 24-fold variation in c-Raf

© 2001 Cancer Research Campaign 

protein levels between the cell lines, both ISIS 5132 and ISIS 13650 substantially reduced cell growth. Most of the cell lines were > 60% growth inhibited, while 3 cell lines (A2780, OVCAR5 and CaOV3) were < 40% growth inhibited by antisense ODNs. These intercellular growth inhibitory differences cannot be accounted for by the variation in c-Raf protein levels between the cell lines. Explanations for these differences are likely to come from further analysis of the growth signalling pathways in these cells. It is also possible that some cell lines do not require c-Raf for cellular proliferation. Such observations have been reported in a study investigating constitutive activation of the ERK signalling pathway in over 100 different tumour cell lines, including OVCAR3, SKOV-3 and OVCAR5 (Hoshino et al, 1999). Both OVCAR3 and SKOV-3 demonstrated high constitutive activation of the ERK pathway and in our study these cell lines were growth inhibited to a great degree by c-Raf antisense ODNs. In contrast, OVCAR5 exhibited minimal constitutive ERK activation that was not increased upon stimulation with serum (Hoshino et al, 1999). This suggests that OVCAR5 may not signal predominantly through c-Raf, and may explain our inability to inhibit OVCAR5 cell proliferation with antisense ODN. Consequently, some ovarian cancer types may be highly responsive to therapies that target c-Raf, while others may not.

Finally, we have demonstrated that ISIS 5132 is effective in vivo, significantly inhibiting the growth of the SKOV-3 xenograft. ISIS 5132 is presently undergoing clinical trials and has been shown to significantly reduce levels of c-raf mRNA in peripheral blood mononuclear cells. This was associated with clinical benefits in 2 out of 14 patients with a mixture of advanced solid tumours (O'Dwyer et al, 1999). ISIS 5132 has been rigorously examined and an antisense mechanism of action has been confirmed (Monia et al, 1996b). Our data further support the validity of ISIS 5132 as an anti-cancer drug that specifically targets c-Raf. In addition we provide evidence that the 2nd generation c-Raf antisense oligonucleotide (ISIS 13650) reduces c-Raf protein levels and inhibits cellular proliferation with potency comparable to ISIS 5132 in vitro.

In summary, our findings demonstrate an association between high levels of c-Raf expression and reduced survival time for patients with ovarian adenocarcinomas suggesting an important role for c-Raf in tumour growth, although the extent of tumour types that heavily depend on c-Raf remains to be explored. We have shown that ODNs that specifically target c-Raf are effective growth inhibitors for the majority of ovarian cancer cells. In addition, the use of antisense ODNs will help identify cells that rely on c-Raf for growth. Studies examining the effects of ISIS 5132 and ISIS 13650 in vivo and in combination with other standard chemotherapeutic agents will give further information on the practical application of antisense ODNs for the treatment of cancer.

### ACKNOWLEDGEMENTS

We thank Dr Jon Holmlund for advice and critical review of the manuscript.

### REFERENCES

Avruch J, Zhang XF and Kyriakis JM (1994) Raf meets Ras: completing the framework of a signal transduction pathway. *Trends Biochem Sci* **19**: 279–283

Cho-Chung YS (1999) Antisense oligonucleotide inhibition of serine/threonine kinases: an innovative approach to cancer treatment. *Pharmacol Ther* **82**: 437–449

Cowsert LM (1997) In vitro and in vivo activity of antisense inhibitors of ras: potential for clinical development. *Anticancer Drug Des* **12**: 359–371

Daum G, Eisenmann-Tapp I, Fries H-W, Troppmair J and Rapp UR (1994) The inns and outs of raf kinases. *TIBS* **19**: 474–480

Fan Z and Mendelsohn J (1998) Therapeutic application of anti-growth factor receptor antibodies. *Curr Opin Oncol* **10**: 67–73

Hoshino R, Chatani Y, Yamori T, Tsuruo T, Oka H, Yoshida O, Shimada Y, Ari-IS, Wada H, Fujimoto J and Kohno M (1999) Constitutive activation of the 41-/43-kDa mitogen-activated protein kinase signaling pathway in human tumors. *Oncogene* **18**: 813–822

Langdon SP, Lawrie SS, Hay FG, Hawkes MM, McDonald A, Hayward IP, Schol DJ, Hilgers J, Leonard RC and Smyth JF (1988) Characterization and properties of nine human ovarian adenocarcinoma cell lines. *Cancer Res* **4**: 6166–6172

Lau QC, Brusselbach S and Muller R (1998) Abrogation of c-Raf expression induces apoptosis in tumour cells. *Oncogene* **16**: 1899–1902

Levack PA, Mullen P, Anderson TJ, Miller WR and Forrest APM (1987) DNA analysis of breast tumour fine needle aspirates using flow cytometry. *Br J Cancer* **56**: 643–646

Meden H and Kuhn W (1997) Overexpression of the oncogene c-erbB-2 (HER2/neu) in ovarian cancer: a new prognostic factor. *Eur J Obstet Gynecol Reprod Biol* **71**: 173–179

Monia BP (1997) First- and second-generation antisense inhibitors targeted to human c-raf kinase: in vitro and in vivo studies. *Anticancer Drug Des* **12**: 327–339

Monia BP, Johnston JF, Geiger T, Muller M and Fabbro D (1996a) Antitumor activity of a phosphorothioate antisense oligodeoxynucleotide targeted against C-raf kinase. *Nature Med* **2**: 668–675

Monia BP, Sasmor H, Johnston JF, Freier SM, Lesnik EA, Muller M, Geiger T, Altmann KH, Moser H and Fabbro D (1996b) Sequence-specific antitumor activity of a phosphorothioate oligodeoxyribonucleotide targeted to human C-raf kinase supports an antisense mechanism of action in vivo. *Proc Natl Acad Sci USA* **93**: 15481–15484

Nemunaitis J, Holmlund JT, Kraynak M, Richards D. Bruce J, Ognoskie N, Kwoh TJ, Geary R, Dorr A, Von Hoff D and Eckhardt SG (1999) Phase I evaluation of ISIS 3521, an antisense oligodeoxynucleotide to protein kinase C-alpha, in patients with advanced cancer. *J Clin Oncol* **17**: 3586–3595

O'Dwyer PJ, Stevenson JP, Gallagher M, Cassella A Vasilevskaya I, Monia BP, Holmlund J, Dorr FA and Yao KS (1999) c-Raf-1 depletion and tumor responses in patients treated with the c-raf-1 antisense oligodeoxynucleotide ISIS 5132 (CGP 69846A). *Clin Cancer Res* **5**: 3977–3982

Rapp UR, Cleveland JL, Bonner TI and Storm SM (1988a) The Raf oncogenes. In: Reddy EP, Skalka AM and Curran T (eds), pp 213–253 The Oncoge ne Handbook,. Amsterdam, the Netherlands. Elsevier Science Publishers B.V. 1988

Rapp UR, Huleihel M, Pawson T, Linnoila I, Minna JD, Heidecker G, Cleveland JL, Beck T, Forchhammer J and Storm SM (1988b) Role of raf oncogenes in lung carcinogenisis. *J Int Assoc Study Lung Cancer* **4**: 162

Rozakis-Adcock M, Fernley R, Wade J, Pawson T and Bowtell D (1993) The SH2 and SH3 domains of mammalian Grb2 couple the EGF receptor to the Ras activator mSos1. *Nature* **363**: 83–85

Stewart CJ, Owens OJ, Richmond JA and McNicol AM (1992) Expression of epidermal growth factor receptor in normal ovary and in ovarian tumors. *Int J Gynecol Pathol* **11**: 266–272

Storm SM, Brennscheidt U, Sithanandam G and Rapp UR (1990) Raf oncogenes in carcinogenesis. *Crit Rev Oncog* **2**: 1–8

Teneriello MG, Ebina M, Linnoila RI, Henry M, Nash JD, Park RC and Birrer MJ (1993) p53 and Ki-ras gene mutations in epithelial ovarian neoplasms. *Cancer Res* **53**: 3103–3108

UKCCCR (1998) United Kingdom co-ordinating committee on cancer research (UKCCCR) Guidelines for the welfare of animals in experimental neoplasia. *Br J Cancer* **77**: 1–10

Witters L, Kumar R, Mandal M, Bennett CF, Miraglia L and Lipton A (1999) Antisense oligonucleotides to the epidermal growth factor receptor. *Breast Cancer Res Treat* **53**: 41–50

Yuen AR, Halsey J, Fisher GA, Holmlund JT, Geary RS, Kwoh TJ, Dorr A and Sikic BI (1999) Phase I study of an antisense oligonucleotide to protein kinase C-alpha (ISIS 3521/CGP64128A) in patients with cancer. *Clin Cancer Res* **5**: 3357–3363

Zheng CF and Guan KL (1993) Properties of MEKs, the kinases that phosphorylate and activate the extracellular signal-regulated kinases. *J Biol Chem* **268**: 23933–23939

© 2001 Cancer Research Campaign

## References

[PDF_00711] Avruch J., Zhang X. F., Kyriakis J. M. (1994). Raf meets Ras: completing the framework of a signal transduction pathway.. Trends Biochem Sci.

[PDF_00714] Cho-Chung Y. S. (1999). Antisense oligonucleotide inhibition of serine/threonine kinases: an innovative approach to cancer treatment.. Pharmacol Ther.

[PDF_00717] Cowsert L. M. (1997). In vitro and in vivo activity of antisense inhibitors of ras: potential for clinical development.. Anticancer Drug Des.

[PDF_00719] Daum G., Eisenmann-Tappe I., Fries H. W., Troppmair J., Rapp U. R. (1994). The ins and outs of Raf kinases.. Trends Biochem Sci.

[PDF_00721] Fan Z., Mendelsohn J. (1998). Therapeutic application of anti-growth factor receptor antibodies.. Curr Opin Oncol.

[PDF_00724] Hoshino R., Chatani Y., Yamori T., Tsuruo T., Oka H., Yoshida O., Shimada Y., Ari-i S., Wada H., Fujimoto J. (1999). Constitutive activation of the 41-/43-kDa mitogen-activated protein kinase signaling pathway in human tumors.. Oncogene.

[PDF_00727] Langdon S. P., Lawrie S. S., Hay F. G., Hawkes M. M., McDonald A., Hayward I. P., Schol D. J., Hilgers J., Leonard R. C., Smyth J. F. (1988). Characterization and properties of nine human ovarian adenocarcinoma cell lines.. Cancer Res.

[PDF_00731] Lau Q. C., Brüsselbach S., Müller R. (1998). Abrogation of c-Raf expression induces apoptosis in tumor cells.. Oncogene.

[PDF_00733] Levack P. A., Mullen P., Anderson T. J., Miller W. R., Forrest A. P. (1987). DNA analysis of breast tumour fine needle aspirates using flow cytometry.. Br J Cancer.

[PDF_00736] Meden H., Kuhn W. (1997). Overexpression of the oncogene c-erbB-2 (HER2/neu) in ovarian cancer: a new prognostic factor.. Eur J Obstet Gynecol Reprod Biol.

[PDF_00739] Monia B. P. (1997). First- and second-generation antisense inhibitors targeted to human c-raf kinase: in vitro and in vivo studies.. Anticancer Drug Des.

[PDF_00741] Monia B. P., Johnston J. F., Geiger T., Muller M., Fabbro D. (1996). Antitumor activity of a phosphorothioate antisense oligodeoxynucleotide targeted against C-raf kinase.. Nat Med.

[PDF_00744] Monia B. P., Sasmor H., Johnston J. F., Freier S. M., Lesnik E. A., Muller M., Geiger T., Altmann K. H., Moser H., Fabbro D. (1996). Sequence-specific antitumor activity of a phosphorothioate oligodeoxyribonucleotide targeted to human C-raf kinase supports an antisense mechanism of action in vivo.. Proc Natl Acad Sci U S A.

[PDF_00749] Nemunaitis J., Holmlund J. T., Kraynak M., Richards D., Bruce J., Ognoskie N., Kwoh T. J., Geary R., Dorr A., Von Hoff D. (1999). Phase I evaluation of ISIS 3521, an antisense oligodeoxynucleotide to protein kinase C-alpha, in patients with advanced cancer.. J Clin Oncol.

[PDF_00753] O'Dwyer P. J., Stevenson J. P., Gallagher M., Cassella A., Vasilevskaya I., Monia B. P., Holmlund J., Dorr F. A., Yao K. S. (1999). c-raf-1 depletion and tumor responses in patients treated with the c-raf-1 antisense oligodeoxynucleotide ISIS 5132 (CGP 69846A).. Clin Cancer Res.

[PDF_00763] Rozakis-Adcock M., Fernley R., Wade J., Pawson T., Bowtell D. (1993). The SH2 and SH3 domains of mammalian Grb2 couple the EGF receptor to the Ras activator mSos1.. Nature.

[PDF_00766] Stewart C. J., Owens O. J., Richmond J. A., McNicol A. M. (1992). Expression of epidermal growth factor receptor in normal ovary and in ovarian tumors.. Int J Gynecol Pathol.

[PDF_00769] Storm S. M., Brennscheidt U., Sithanandam G., Rapp U. R. (1990). raf oncogenes in carcinogenesis.. Crit Rev Oncog.

[PDF_00771] Teneriello M. G., Ebina M., Linnoila R. I., Henry M., Nash J. D., Park R. C., Birrer M. J. (1993). p53 and Ki-ras gene mutations in epithelial ovarian neoplasms.. Cancer Res.

[PDF_00777] Witters L., Kumar R., Mandal M., Bennett C. F., Miraglia L., Lipton A. (1999). Antisense oligonucleotides to the epidermal growth factor receptor.. Breast Cancer Res Treat.

[PDF_00780] Yuen A. R., Halsey J., Fisher G. A., Holmlund J. T., Geary R. S., Kwoh T. J., Dorr A., Sikic B. I. (1999). Phase I study of an antisense oligonucleotide to protein kinase C-alpha (ISIS 3521/CGP 64128A) in patients with cancer.. Clin Cancer Res.

[PDF_00784] Zheng C. F., Guan K. L. (1993). Properties of MEKs, the kinases that phosphorylate and activate the extracellular signal-regulated kinases.. J Biol Chem.

